# Mass drug administration campaigns: comparing two approaches for schistosomiasis and soil-transmitted helminths prevention and control in selected Southern Malawi districts

**DOI:** 10.1186/s12913-023-10489-5

**Published:** 2024-01-03

**Authors:** Peter Makaula, Sekeleghe Amos Kayuni, Kondwani Chidzammbuyo Mamba, Grace Bongololo, Mathias Funsanani, Lazarus Tito Juziwelo, Janelisa Musaya, Peter Furu

**Affiliations:** 1 Research for Health Environment and Development, P.O. Box 345, Mangochi, Malawi; 2 Malawi Liverpool Wellcome Research Programme, Private Bag 30096, Blantyre 3, Malawi; 3 Medical Aid Society of Malawi (MASM) Medi Clinics Limited, Area 12 Medi Clinic, P.O. Box 31659, Lilongwe 3, Malawi; 4 Liverpool School of Tropical Medicine, Pembroke Place, Liverpool, L3 5QA UK; 5 Mangochi District Council, District Health Office, P.O. Box 42, Mangochi, Malawi; 6 Ministry of Health, Community Health Sciences Unit, National Schistosomiasis and Soil-Transmitted Helminths Control Programme, Private Bag 65, Lilongwe, Malawi; 7 Department of Pathology, Kamuzu University of Health Sciences, Private Bag 360, Blantyre 3, Malawi; 8 Department of Public Health, Global Health Section, University of Copenhagen, 5 Øster Farimagsgade, 1014 Copenhagen K, Denmark

**Keywords:** Mass drug administration, Community-directed intervention, Neglected tropical diseases, Schistosomiasis, Soil-transmitted helminths

## Abstract

**Background:**

Mass drug administration is one of the key interventions recommended by WHO to control certain NTDs. With most support from donors, health workers distribute antihelminthic drugs annually in Malawi. Mean community coverage of MDA from 2018 to 2020 was high at 87% for praziquantel and 82% for albendazole. However, once donor support diminishes sustaining these levels will be challenging. This study intended to compare the use of the community-directed intervention approach with the standard practice of using health workers in delivery of MDA campaigns.

**Methods:**

This was a controlled implementation study carried out in three districts, where four health centres and 16 villages in each district were selected and randomly assigned to intervention and control arms which implemented MDA campaigns using the CDI approach and the standard practice, respectively. Cross-sectional and mixed methods approach to data collection was used focusing on quantitative data for coverage and knowledge levels and qualitative data to assess perceptions of health providers and beneficiaries at baseline and follow-up assessments. Quantitative and qualitative data were analyzed using IBM SPSS software version 26 and NVivo 12 for Windows, respectively.

**Results:**

At follow-up, knowledge levels increased, majority of the respondents were more knowledgeable about what schistosomiasis was (41%-44%), its causes (41%-44%) and what STH were (48%-64%), while knowledge on intermediate host for schistosomiasis (19%-22%), its types (9%-13%) and what causes STH (15%-16%) were less known both in intervention and control arm communities. High coverage rates for praziquantel were registered in intervention (83%-89%) and control (86%-89%) communities, intervention (59%-79) and control (53%-86%) schools. Costs for implementation of the study indicated that the intervention arm used more resources than the control arm. Health workers and community members perceived the use of the CDI approach as a good initiative and more favorable over the standard practice.

**Conclusions:**

The use of the CDI in delivery of MDA campaigns against schistosomiasis and STH appears feasible, retains high coverages and is acceptable in intervention communities. Despite the initial high costs incurred, embedding into community delivery platforms could be considered as a possible way forward addressing the sustainability concern when current donor support wanes.

**Trial registration:**

Pan-African Clinical Trials Registry PACTR202102477794401, date: 25/02/2021.

**Supplementary Information:**

The online version contains supplementary material available at 10.1186/s12913-023-10489-5.

## Background

Malawi is endemic for several neglected tropical diseases (NTD) and since 1994 studies have shown schistosomiasis and soil-transmitted helminths (STH) as of major public health importance [1]. These diseases require special attention due to their high public health significance and varied mass drug administration (MDA) coverage. MDA is one of the community-based programmes which in Malawi, is widely carried out annually to prevent and control schistosomiasis and STH [2, 3]. During MDA campaigns, it is mostly the Health Surveillance Assistants (HSAs) who are the community-based health workers responsible for the distribution of medicine. During MDA campaigns carried out in years, 2018 to 2020, community coverage of MDA in Malawi was high at 87% (range 51.5%-95.0%) for praziquantel and 82% (range 30.6%-92.3%) for albendazole mainly due to donor support by Unlimit Health (formerly Schistosomiasis Control Initiative Foundation), and other partners [3, 4]. However, the existing treatment approach represents a challenge for maintaining a high, sustainable level of coverage and uptake once donors’ support is reduced.

Evidence points to great successes in control of helminthic NTDs achieved through use of community-based interventions such as community-directed treatment (ComDT) and community-directed intervention (CDI) compared to use of routine health facility based or no intervention [5–8]. This is exemplified in the case of Burkina Faso, the first country in the WHO African Region to achieve nationwide coverage with anthelminthic drugs for three major NTDs namely, lymphatic filariasis, STH and schistosomiasis. Here community-based interventions have achieved high coverage without any increase in implementation costs at district and health facility levels [9].

Community-directed intervention is defined as a health intervention that is undertaken by community implementers under the direction of the community itself [5]. The approach has been used successfully to distribute vitamin A and long-lasting insecticide treated nets as well as in home management of malaria [5]. The CDI strategy has also been implemented to control schistosomiasis and STH in Cameroon [10, 11], Kenya [12, 13], Malawi [14, 15], Mali [16], Nigeria [17] and Uganda [18, 19]. To the best of our knowledge, no study has tested the use of the CDI approach to deliver MDA campaigns for control of schistosomiasis and STH in Malawi. In this project, we hypothesized that the combination of publicly prioritized MDA efforts, a successful CDI programme and a well-organized community set-up would provide synergy and increased empowerment, efficiency, coverage, health impact and sustainability for MDA. This study, therefore, intended to compare the use of the CDI approach with the standard practice of using community-based health workers in delivery of MDA campaigns against schistosomiasis and STH in selected districts of southern Malawi. The selection of schistosomiasis in this study was of our interest because its drug, praziquantel requires calculation of specific dosage before administration to a person and due the associated adverse effects which require proper observation and management.

## Methods

Reporting of this study has been verified in accordance with the strengthening the reporting of observational studies in epidemiology (STROBE) checklist (see Additional file 1) [20].

### Study design

This study was designed as a controlled implementation research with two arms, namely, an intervention arm – which implemented MDA campaigns at community level using the study-directed CDI approach of using community-based volunteers, and a control arm – the standard practice, which implemented no project-directed MDA campaigns but relied on routine campaigns of using community-based health workers organized by the National Schistosomiasis and STH Control Programme.

### Study area, target population and sample sizes

**Fig. 1:**
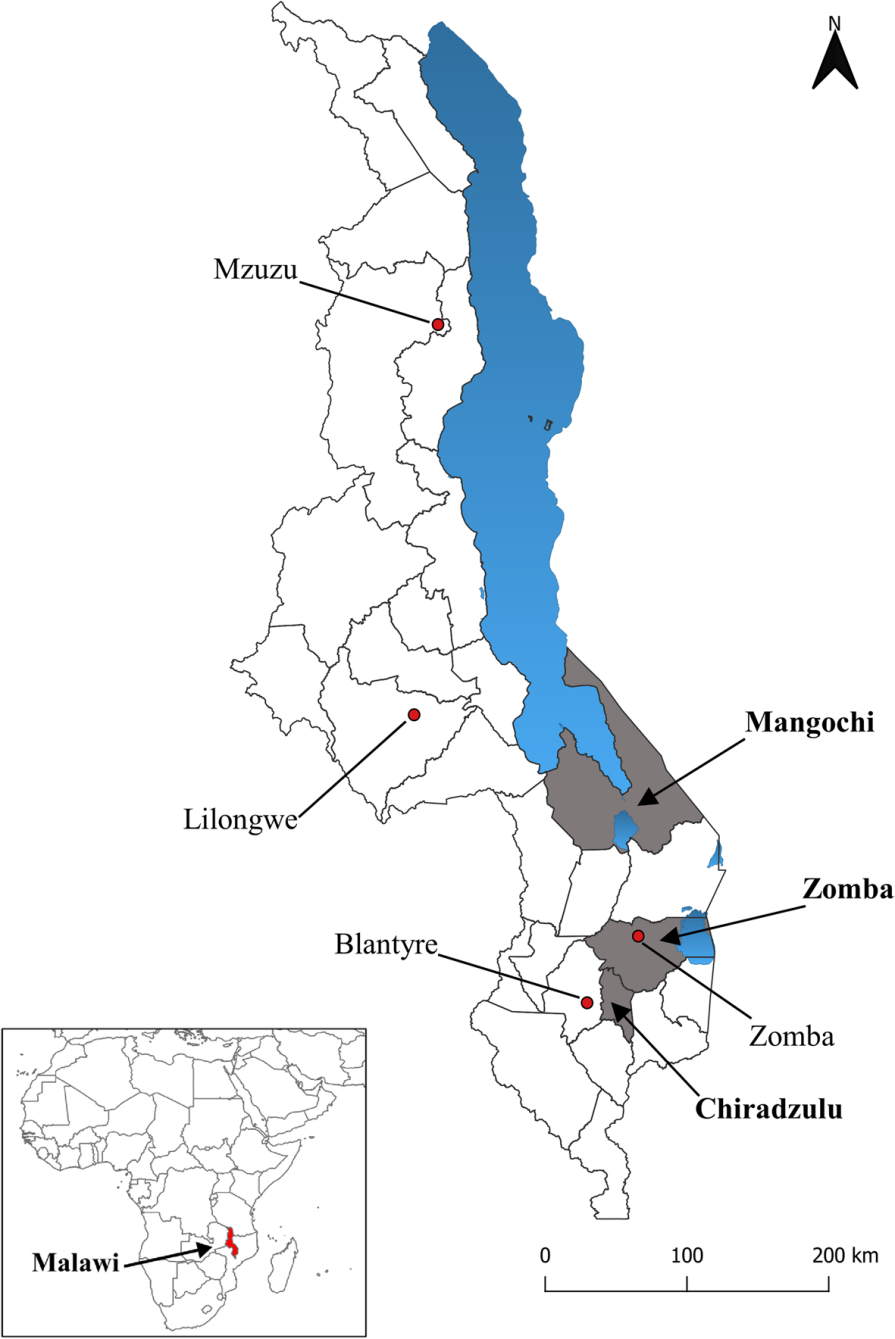
Locations of the districts of Mangochi, Zomba and Chiradzulu (in gray), Lake Malawi and others small lakes (in blue), major cities of Mzuzu, Lilongwe, Zomba and Blantyre and the location of Malawi in Africa (red in the inset) (Source: Authors’ own map [4, 14, 15])

The study was carried out in the three southern Malawi districts of Chiradzulu, Mangochi and Zomba (Fig. 1).

The study districts were selected purposively based on their level of co-endemicity with schistosomiasis and STH, and the comparative socio-economic, demographic and health indicators of the three districts [2]. In each district, four health centres and 16 villages within the catchment areas of the health centres were randomly selected to participate in the study [4]. Each study arm was randomly assigned two health centres with eight villages in total (see Additional file 2) and the demographic and socio-economic characteristics of the respondents for the three study districts (Additional file 3).

The study population comprised the District NTD Coordinators, Pharmacy Technicians, representatives of implementation partners involved in delivery of MDA for schistosomiasis and STH, officers in-charge (clinicians or nurses), HSA, community leaders and adult community members aged 15 years or above. Sampling techniques deployed at district level included purposive selection of key informants namely, the District NTD Coordinators, Pharmacy Technicians, and representatives of some of the partners involved in delivery of MDA for schistosomiasis and STH in each district for face-to-face in-depth interviews [4]. At health centre level, clinicians or nurses in-charge or their representatives, and Senior HSA were purposively selected to participate in the study. At community level, we purposively selected responsible HSA and community leaders from the targeted villages while taking into consideration their diverse gender and roles. In addition, to obtain a varied community representation and a detailed impression of community perceptions, we randomly invited different homogenous groups of eight to ten people from selected villages to participate in a focus group discussion (FGD). Lastly, in every village a predetermined number of households were randomly selected to participate in a face-to-face questionnaire-based knowledge, attitudes, and practices (KAP) survey. Any household member aged 15 years or above available during the time of the visit was invited to participate in the survey while ensuring gender balance [4]. Table 1 summarizes the methods, purposes, levels, sample sizes and quantities of data collected in the study.

**Table 1:** Methods, purposes, levels, sample sizes and amount of data collected in the study

Methods (BL/FU)^a^	Purpose of data collected	Data collection—levels and numbers collected					Totals
		District	Implementation partners	Health Centre	Village	Household	
1. Questionnaire surveys	Knowledge, attitudes and practices	-	-	-	-	379/382	379/382
2. In-depth interviews	Process/perceptions/benefits/critical factors	6	3	12	41	-	62
3. Health Management and Information System	Coverage/disease burden	3/3	-	-	-	-	3/3
4. Focus group discussions	Perceptions/benefits/critical factors	-	-	-	12	-	12

^a^BL/FU = Baseline (May 2020) [4]/Follow-up (May 2021)

### The process of using the CDI approach to deliver MDA campaigns

In the intervention arm, at community level volunteers were mobilized and sensitized on how to direct and implement MDA campaigns using the CDI approach. The CDI training guidelines and materials were adapted from the WHO [21] and the Johns Hopkins Program for International Education in Gynaecology and Obstetrics (JHPIEGO) [22]. The processes that were used to introduce CDI in the study are described as follows:

#### At district level

Identification of implementation partners was done, and they included Nutrition and Access to Primary Education in Chiradzulu district, Save the Children in Zomba district, and Blantyre Institute of Community Outreach in Mangochi.

The District Health Management Team selected and endorsed the District Environmental Health Officer, the District NTD Coordinator, a Community Health Nurse and a Pharmacy Technician as trainers and supervisors to implement this study. These were trained by the research team on the overall aims of the study, principles and process of the CDI approach, and on available interventions of the study. This team was responsible for training health centre-based health workers who comprised either a Medical Assistant or a nurse in charge, a Senior HSA and an HSA responsible for each of the four participating villages under the intervention arm of the study. It was the health centre-based workers who trained and supervised the community-based volunteers known as community-directed distributors (CDDs).

#### At health centre and village levels

Trained health centre-based staff together with the HSAs responsible for the villages conducted community meetings from where CDDs or volunteers were identified (one volunteer per 200 people considering literacy and gender factors). The volunteers were trained and assigned roles as CDDs of the selected interventions with continued supervision from the health centre-based staff throughout the study implementation period.

At every stage at health centre and village levels, both the district and health centre teams participated in the training as observers to ensure quality delivery and adherence to the study protocol. In the control arm of the study, no briefing and training were offered to health staff from the corresponding health centres and villages.

#### Roles and responsibilities of the key players in CDI process

During the implementation of the CDI process at community level, the health services, implementation partners and the community had the following roles and responsibilities (Table 2).

**Table 2:** Roles and responsibilities of the key players in CDI process

Category of players	Roles and responsibilities played in CDI process
1) District-level officers	• Introduced to the community the concept of CDI and technical aspects of the interventions • Provided and facilitated capacity building, supplies and technical support as required by the interventions • Provided and supported supervision to health centres based on procedures and criteria of the interventions
2) Implementation partners	• Provided and facilitated capacity-building and technical support as required by the interventions • Provided and supported supervision on the basis of procedures and criteria of the interventions that were agreed upon with the community • Ensured adequate provision of the necessary supplies i.e., drugs and other intervention materials
3) Health centre staff and responsible HSAs	• Identified community leadership structures and socio-cultural organizations and took these into account in all interactions with the community • Introduced to the communities the concept of CDI and technical aspects of the interventions • Provided and supported supervision to Village Health Committees, CDDs and communities based on procedures and criteria of the interventions
4) Community members	With the facilitation by the health centre staff and responsible HSAs, community members had the following roles and responsibilities: • Collectively discussed the health problems and possible interventions from their own perspective while considering relevant community knowledge and information provided to them by the health professionals • Collectively decided whether they will take responsibility for implementation of interventions at community level • Collectively identified volunteers to serve as CDDs • Collectively agreed on the approach to implementing the interventions in their communities • Collectively designed the approach to implementing the interventions in their communities • Collectively identified the required resources from within their communities • Collectively planned how, when, where and by whom to implement the interventions • Collectively supervised and decided on what support to be provided to CDDs and how to monitor the processes • Executed the interventions (mainly by CDDs) • Collectively reviewed the implementation process where necessary

### Data collection 

A mixed methods approach to data collection during baseline [4] and follow-up assessments was done focusing on quantitative data for coverage and cost estimates, and qualitative data for assessing perceptions of health providers and beneficiaries and evaluating processes regarding interventions. Cross-sectional data collection methods were used during baseline [4] and follow-up studies where all the necessary data for the study were similarly collected from intervention and control arms during baseline and follow-up assessments. Data were collected from the involved health professionals, implementation partners, community leaders, MDA implementers and community members as beneficiaries using data collection instruments previously used and published by the research team [4, 14, 15]. As part of implementation of MDA, behavior change communication messages for schistosomiasis and STH prevention and control were also delivered to bring about positive changes in knowledge regarding the diseases and MDA amongst people residing in the communities. To assess levels of knowledge changes, the study conducted two population-based questionnaire surveys in May 2020 as baseline [4] and in May 2021 as follow-up for intervention and control arms. A questionnaire that was programmed in tablets [4] was administered to adult household representatives at community level for determining respondents’ knowledge, attitudes and practices regarding control and prevention of schistosomiasis and STH, and delivery of MDA. Moreover, schistosomiasis and STH treatment records for preceding years of October 2019 (baseline) [4] and October 2020 (follow-up) were obtained and reviewed using checklists to establish MDA coverage data at district, health centre and village levels.

Qualitative data collection instruments comprised in-depth interview guides previously used by the research team [15] were administered to NTD Coordinators and health professionals at district and health centre levels, HSAs, and leaders at community level, to evaluate the processes used during interventions delivery and for determining perceptions of health providers and beneficiaries, benefits, and critical factors. Similarly, focus group discussion guides [15] were used to conduct group interviews with beneficiaries about their perceptions on using the interventions and benefits. All the proceedings of the key informant interviews and FGDs were recorded using digital audio recorders. Finally, document reviews were carried out to get an insight on the national prescription of health policy, priority health issues, coverage, costs, strategy and effectiveness of MDA delivery, availability of resources for MDA and the existing challenges and opportunities. Data were collected in three districts comprising totals of 12 health centres and 48 villages during the month of May for baseline (2020) [4] and for follow-up (2021) surveys.

### Data management and analyses

Quantitative data collected through survey questionnaires was programmed in tablets using the Secure Data Kit [23]. Questionnaire and checklists data were processed and analyzed using IBM Statistical Package for Social Sciences (SPSS) software version 26. Analysis involved calculation of percentages, tabulations, and frequencies to estimate MDA coverage. Furthermore, statistical significance tests using Chi Square were performed on differences in delta values (i.e., differences between baseline and follow-up) for MDA coverage for praziquantel (schistosomiasis) and albendazole (STH) between intervention and control groups. The analyses of costs and benefits data were carried out using procedures outlined by Makaula et al. (2019) [15]. Qualitative data consisted of textual and audio data, including transcripts of key informant interviews, transcripts of FGDs, field notes on observations and other intervention-specific insights, notes, and reports from meetings. Transcripts were translated into English. A computer-assisted qualitative content analysis of the data using NVivo 12 for Windows (QSR International), a qualitative data analysis software programme. Data were analyzed using open coding to come up with cross-classification and retrieval of categories of texts by theme.

## Results

### Comparison of schistosomiasis and STH knowledge levels between intervention and control arms 

**Fig. 2:**
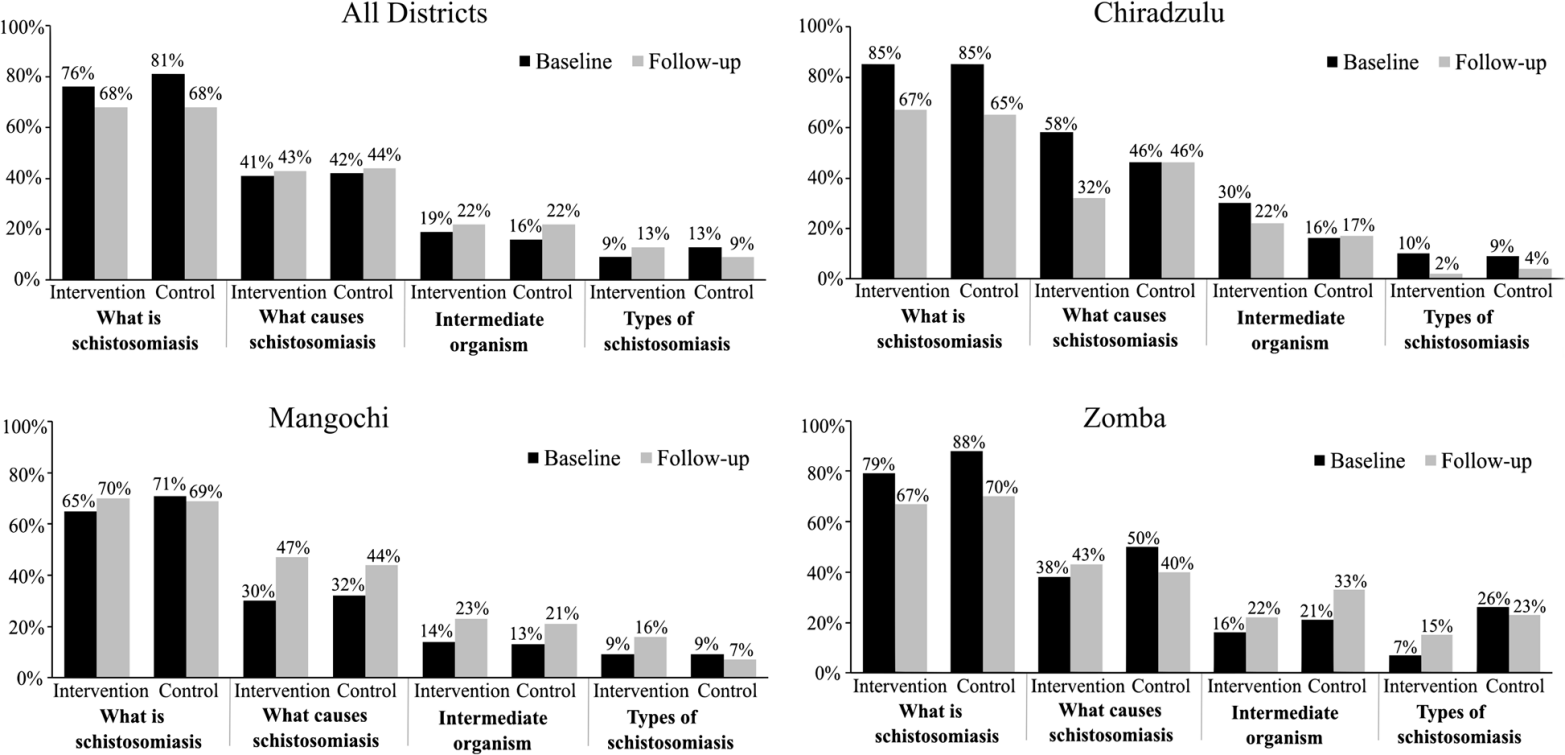
Knowledge of schistosomiasis between intervention and control arms in all districts, Chiradzulu, Mangochi and Zomba

A comparison between baseline and follow-up surveys results revealed that knowledge levels about schistosomiasis and STH varied both in intervention and control arms across the study districts. Majority of the respondents are more knowledgeable about what schistosomiasis is during baseline and follow-up both in intervention and control communities. However, respondents’ knowledge on causes of schistosomiasis, its intermediate host and its types were less known or understood as few people gave the correct answers. In all the districts, the baseline and follow-up results showed that knowledge levels increased for causes of schistosomiasis, name of the intermediate organism for schistosomiasis for intervention and control arms, and types of schistosomiasis for intervention arm. Among the districts, Zomba was highest in terms of knowledge levels, followed by Chiradzulu and Mangochi. Among the three districts, general knowledge levels about schistosomiasis were 9%-81% at baseline which declined to 9%-68% at follow-up for both in intervention and control arms but not statistically significant (p 0.67) (Fig. 2).

**Fig. 3:**
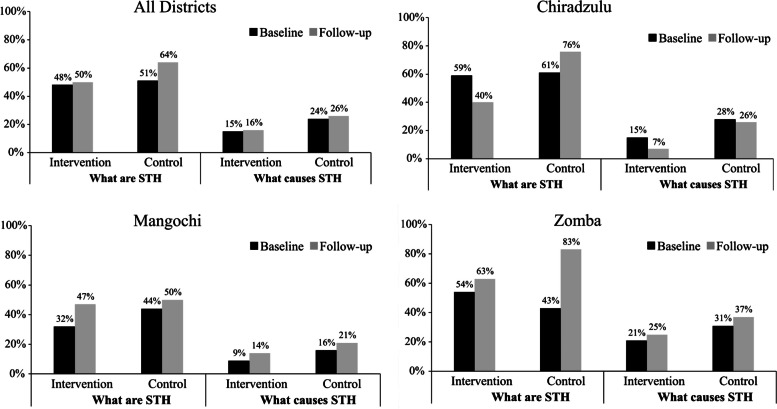
Knowledge of soil-transmitted helminths between intervention and control arms in all Districts, Chiradzulu, Mangochi and Zomba

With regards to knowledge of STH, a comparison between baseline and follow-up results revealed that communities in intervention and control arms in the districts have varying understanding of the diseases. The baseline and follow-up surveys revealed that most of the respondents are knowledgeable about what STH are, both in intervention and control communities. However, very low knowledge levels were obtained when respondents were asked to mention what causes STH. In all districts, knowledge levels increased for all two indicators on what STH are and causes of STH for intervention and control arms. In the districts, Zomba was highest in terms of knowledge levels, followed by Chiradzulu and Mangochi. Among the three districts, general knowledge levels about STH were 15%-51% at baseline which significantly increased to 16%-64% at follow-up for both in intervention and control arms (p 0.04) (Fig. 3).

### Comparison of MDA coverage trends for praziquantel and albendazole in study districts

**Fig. 4:**
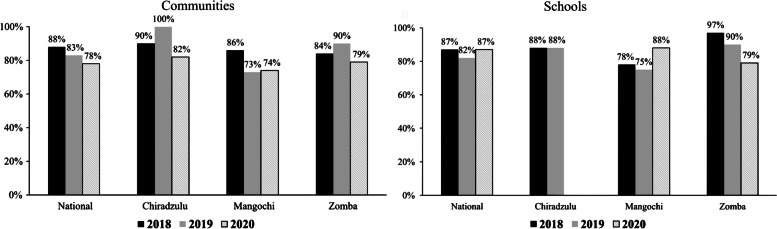
Praziquantel coverage trends in Communities and Schools for study districts over three years

The study targeted delivery of MDA campaigns in 2018, 2019 and 2020 using praziquantel for schistosomiasis and albendazole for STH. The MDA deliveries for these years were done by HSAs in all the districts, except for the 2020 MDA in the intervention communities which used the CDI approach by CDDs. MDA data for praziquantel and albendazole for the three years were analyzed for comparison of coverage trends. Comparison of praziquantel coverage rates during the years revealed that all the districts registered high coverage rates for praziquantel using community-based MDA at 83% (range 73%-100%) and school-based MDA at 87% (range 75%-92%). For praziquantel community-based MDA, Chiradzulu district scored highest rates in the three years. As for praziquantel school-based MDA, Zomba district was highest in 2018 and 2019, while Mangochi district came highest in 2020. No praziquantel MDA was carried out in Chiradzulu schools 2020 due to COVID-19 schools’ closure in the district. Figure 4 illustrates praziquantel coverage trends for the study districts during the three years.

**Fig. 5:**
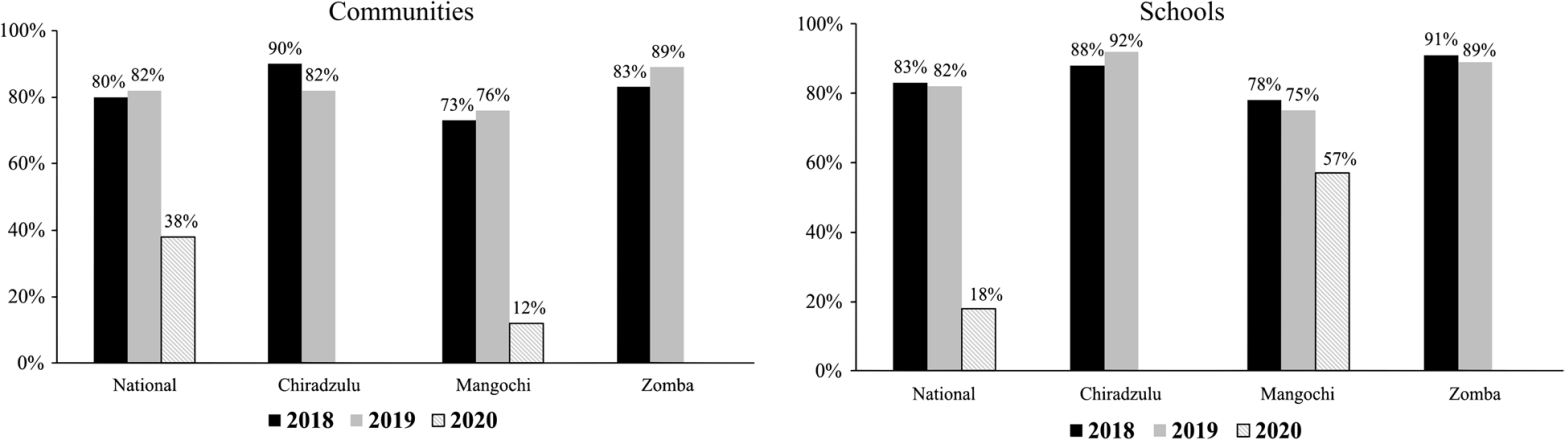
Albendazole coverage trends in Communities and Schools for study districts over three years

A comparison between albendazole MDA coverage rates for 2018 and 2019 revealed that there were no differences among the districts in terms of distribution although the 2019 rates were higher than those obtained in 2018. No differences were also observed for both albendazole coverage for community and school modes of MDA deliveries. High coverage trends for community and for schools were observed in albendazole MDA in all the districts. In 2018 and 2019, Chiradzulu (90%) and Zomba (90%) respectively had highest albendazole coverage using the community MDA delivery approach. As for school coverage, Zomba was highest for both 2018 and 2019. In 2020, there was no albendazole MDA done for communities and schools in the study districts except for Mangochi which registered very low coverage rates in communities (12%) and schools (57%) due to a logistical supply chain problem. Figure 5 illustrates albendazole coverage trends for the study districts during the three years.

### Comparison of coverage for praziquantel in study districts by arms of study

Comparisons were made between intervention and control arms of the study for baseline and follow-up praziquantel coverage data in the three districts. Generally, in all the districts increases in coverage rates were registered in intervention (from 83 to 89%) and control (from 86 to 89%) communities, while decreases were recorded for intervention (from 79 to 59%) and control (from 86 to 53%) schools.

In Chiradzulu district, there were decreases in praziquantel MDA coverage for intervention (from 100 to 91%) and control (from 100 to 93%) communities. Despite registering high praziquantel MDA coverage rates in intervention (88%) and control (92%) schools during baseline, there was no follow-up MDA done due to schools’ closure brought about by the coronavirus disease 2019 (COVID-19) pandemic. There were no observed differences between coverage scores in intervention and control arms for both community and school MDA in the district.

In Mangochi district, follow-up MDA coverage rates were higher than those obtained in baseline for both community and school praziquantel MDA. Increases in coverage rates were observed in intervention (from 62 to 96%) and control (from 75 to 99%) communities; and in intervention (from 61 to 94%) and control (from 83 to 85%) schools. There were no observed differences between follow-up coverage rates in intervention and control arms and for communities and schools.

**Fig. 6:**
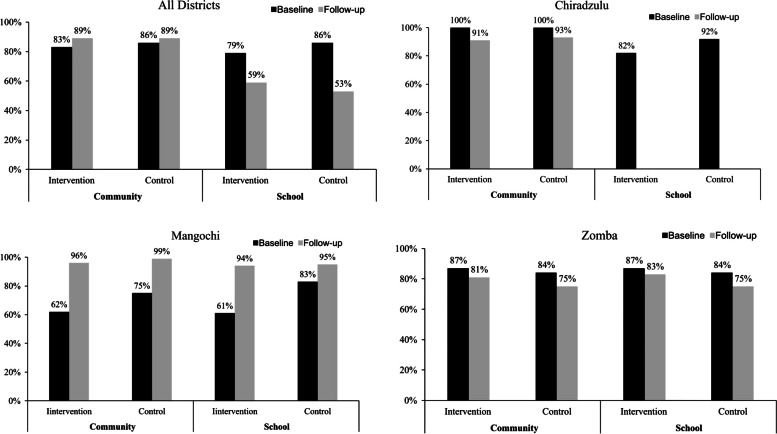
Praziquantel coverage by arms of study in all districts, Chiradzulu, Mangochi and Zomba

Praziquantel coverage rates in Zomba were low during follow-up than during baseline in both communities and schools. The coverage rates decreased in intervention communities (from 87 to 81%) and schools (from 87%—83%); and control communities and schools (from 84 to 75% for both). However, there were no observed differences between coverage scores between intervention and control arms for both communities and schools meaning that there was no effect on the follow-up MDA attributable to the implementation of the study in the district. Among the two districts of Mangochi and Zomba, praziquantel coverage were 79%-86% at baseline which increased for communities and decreased for schools 53%-89% at follow-up for both in intervention and control arms but not statistically significant (p 0.82) (Fig. 6).

### Statistical significance of the MDA coverage difference at follow-up stage, in intervention and control groups

**Fig. 7:**
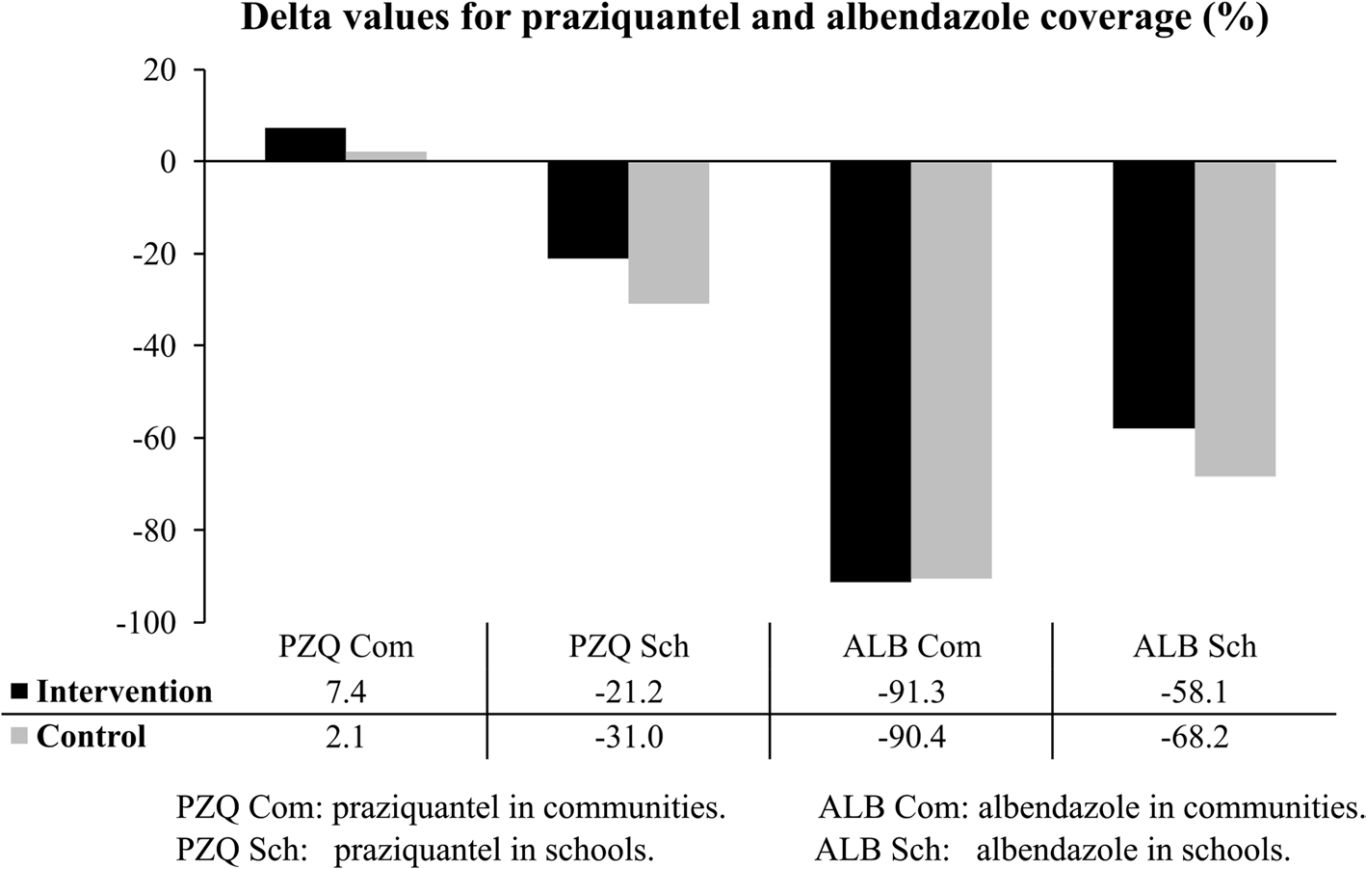
Differences in average coverage rates (%) for praziquantel and albendazole between Baseline and Follow-up for intervention and control communities and schools

When overall differences (delta values) between baseline and follow-up coverage rates in all the districts were calculated for praziquantel and albendazole in each study arm, it was observed that increases were only registered for praziquantel in communities in the intervention (7.4%) and control (2.1%) arms. Decreases were observed for distribution of praziquantel in schools for intervention (-21.2%) and control (-31%) arms for Mangochi and Zomba only. As for distribution of albendazole follow-up for Mangochi only, decreases were noted in communities for intervention (-91.3%) and control arms (-90.4%), and albendazole in schools for intervention (-58.1%) and control arms (-68.2%). These decreases were higher in the control arm for distribution of praziquantel in schools (-31%) and albendazole in schools (-68.2%) while for distribution of albendazole in communities the decrease was higher in the intervention arm (-91.3%). Chi Square test conducted for each MDA component showed that differences in delta values in intervention and control arms were not statistically significant (p 0.14) for all the components (Fig. 7).

### Comparison of costs for using the CDI approach and the standard practice to deliver MDA campaigns

Cost data for implementation of the two approaches at three levels of study implementation: district, health centre and community were compiled and analyzed. Cost data used for comparative analysis included four main items on personnel costs of health worker and volunteer allowances during study activities, transportation, communication, and other logistics such COVID-19 preventive supplies, stationery, refreshments. Out of the total amount of resources used to directly implement the selected study activities at district, health centre and community levels, most resources were used for personnel related costs (85%) followed by other logistics (9%) and transportation (5%). For both arms of the study (intervention and control), most resources were used at community level (51%) followed by health centre level (29%) and district level (19%). At both health centre and community levels, the intervention arm used more resources at 27 percent and 44 percent than the control arm at 2 percent and 4 percent respectively (Table 3).

**Table 3:** A summary of direct costs of implementing CDI and standard practice to deliver MDA in the study districts

Cost items	Estimated costs according to level and arm used converted to US$^a^					Total US$ for cost item (% of total)
	District	Health centre		Community		
		Intervention	Control	Intervention	Control	
1. Personnel	1,969.73	4,637.13	435.39	8,314.51	1,421.56	16,778.33 (85%)
2. Transportation	652.49	250.48	-	-	-	902.97 (5%)
3. Communication	118.03	39.34	-	-	-	157.37 (1%)
4. Other logistics	1,074.04	409.35	-	365.88	-	1,849.28 (9%)
Totals for level/arm (% of total)	3,814.29 (19%)	5,336.30 (27%)	435.39 (2%)	8,680.40 (44%)	1,421.56 (7%)	19,687.95 (100%)

^**a**^Average exchange rate: 1 United States Dollar = 762.54 Malawi Kwacha

### Perceptions of health workers and community members on the use of the CDI approach in MDA delivery

The study sought to get the perceptions of the health workers as providers and community members as beneficiaries in the intervention arm on the use CDDs in delivery of MDA. Most of the health providers at district and facility levels perceived the use of the CDI approach to deliver MDA as a welcomed and a good initiative.

“I think drugs should be administered by the communities themselves, because these people reside in the same area, and they know their fellow community members very well as compared to health workers.”—A male health worker, Chiradzulu.

On the other hand, majority of the community leaders and members viewed the CDI approach convenient because people were easily accessing drugs right in their homes.

"It is a good development, largely because the community feels free to talk with the volunteers because they are part of them unlike the HSAs who are like strangers in the community.”—KII Community leader, Mangochi.

These sentiments were shared by the CDDs who expressed satisfaction with their roles.

“It’s much easier for people to get medication from us because I come from the same village. People may spend more money when they go to the health centre while as within the village people will get services while doing their work at home.” – KII female CDD, Chiradzulu.

However, there were mixed views on the need for giving some incentives to CDDs, especially about what kind of incentives and who should be responsible for them between the community and government.

*“It is good* (incentives) *since it acts as one way of motivation, however, on the other hand it is not good because when people are used to incentives, they are less likely to participate in the event that an activity does not have incentives.”* – KII male partner, Zomba.

### Critical factors for effective implementation and sustainability of using the CDI approach for delivery of MDA campaigns

An analysis of qualitative interviews data according to the health officials, partners and community members identified several critical factors for effective implementation and sustainability of using the CDI approach for delivery of MDA campaigns. There is a need for engaging stakeholders at all levels to make them understand and appreciate the relevance of the approach as an alternative mode of delivering health services, empowering communities to promote local participation and ownership of the entire implementation process. There is also a need for an enabling environment for applying the CDI approach in terms of securing supportive policies, availability of supplies, committed staff and proper and timely management of adverse effects of drugs. Selection of appropriate, willing, and motivated individuals with requisite skills, trusted to serve as CDI implementers. Lastly, use of incentives or rewards to community volunteers during delivery of health services generated mixed views amongst stakeholders. While some stakeholders applauded giving of incentives to the volunteers arguing that incentives encourage hard work, others completely disagreed with the idea noting that they may defeat the whole notion of community ownership and sustainability which are vital tenets of the CDI approach.

## Discussion

The results of this study have revealed that knowledge levels in relation to schistosomiasis and STH varied disproportionately according to disease aspects asked about for both intervention and control arms across the districts. The findings agree with other studies done in Malawi [4], Philippines [24], Nigeria [25], Egypt [26], Cameroon [27], Papua New Guinea [28] and Turkey [29] demonstrating that despite implementation of numerous activities towards the control of NTDs, there was little sensitization of the public to increase awareness of the diseases.

Achieving high treatment coverage depends on the willingness of the community to participate in MDA programmes. It therefore important that issues that can hinder community engagement such as lack of awareness, misconceptions, or mistrust should be adequately addressed through intensified community sensitization and health education programmes that involve the beneficiaries, community volunteers, local leaders, and health workers to build trust and increase awareness.

The study’s findings have also revealed that the trends for MDA coverage rates for praziquantel and albendazole from 2018 to 2020 were high in the three study districts and that there were no differences observed between coverage trends for praziquantel and albendazole, nor for communities and schools [4]. These high rates trends obtained in the districts during the three years were like the national average coverage rates indicative that as a country, Malawi performance is satisfactory and probably on the right path towards the goal of reducing schistosomiasis and STH as public health problems [4, 30]. There are though some variations observed on how the individual districts and study arms performed. These variations observed between intervention and control communities in follow-up when compared to those obtained in baseline mean that implementation of the CDI approach did not have a positive impact on coverages. The observed variations in coverage results might have been influenced by other intra district-specific organizational factors than those attributed to implementation of the study. The study findings have furthermore shown that during follow-up evaluation there were no observed differences in praziquantel and albendazole MDA coverage rates between intervention and control arms for both communities and schools. These high MDA coverage rates are in consistent with what other recent studies carried out in Philippines [24], Ghana [31], Cote d’Ivoire, Kenya, Mozambique, Niger and Tanzania [32, 33], Sierra Leone [34], Togo [35] and Zanzibar [36]. A spatiotemporal modelling review [37] has also reported that schistosomiasis prevalence in sub-Saharan Africa has decreased considerably, most likely explained by the scale-up of preventive chemotherapy. Prevalence studies done in several districts including in the current study districts of Zomba and Mangochi have pointed to the possibility of persistent infections, reinfections, and drug resistances after introduction of MDA in 2009 occurring within a year despite sustained annual MDA campaigns implemented throughout the country [4] which are retarding the prospects of attaining the set goal of reducing the burden of schistosomiasis and STH to levels of no public health importance by 2030. These persistent infections, reinfections and drug resistances are like those observed in other studies carried out elsewhere in other sub-Saharan Africa countries [35, 38].

It was noted that the delta values had increases only for distribution of praziquantel in communities for the intervention and control arms, while various degrees of decreases occurred for praziquantel in schools in the intervention and control arms, and for all distributions of albendazole in schools and communities for both intervention and control arms. These observed decreases are contrary to what was hypothesized in the study for increased coverage rates in the intervention communities as compared to control communities. These findings again, demonstrate that there was no positive impact attributable to the implementation of the study apart from demonstrating the feasibility of using the CDI approach to deliver MDA campaigns in the study districts. We believe that since this was first time the CDI approach was used in Malawi for delivery of MDA campaign for praziquantel and albendazole, there is a likelihood that the coverage rates may increase with subsequent implementation because CDDs will now be more familiar with the process. A major contributing factor for variations in coverage rates that were obtained in this study was due to emergence of the COVID-19 pandemic that was a stress factor for the health system and might have influenced compliance and uptake of MDA in both intervention and control communities. The decreases related to distribution of albendazole were mostly due stock outs of drug stocks at national level during the 2020 MDA campaign and the few that were treated used districts’ drug balances from the previous 2019 MDA campaign. Previous studies which were carried out elsewhere in Kenya [12, 13], Malawi [15], Mali [16], Nigeria [17] and Uganda [18, 19] reported higher treatment coverage rates in areas where CDI was used than where it was not used. We attribute the slightly different findings obtained in this study to the fact that the study was conducted under the COVID-19 pandemic condition which might also have negatively influenced the results due to misinformation associated to distribution of drugs or vaccine in communities. Selection of already high coverage areas to participate in the study also obscured the likelihood of registering positive impact for implementing the study.

A comparative analysis of the cost data for implementation of the two approaches has revealed that the intervention arm used more resources (about eight times) than the control arm. Generally, the implementation of the study incurred more resources than originally planned due to the emergence of the COVID-19 pandemic. The study incurred additional costs related to procurement of COVID-19 personal protective equipment for use by the researchers, participants, and trainees in the various planned activities. Duplication of activities were also made in some instances to minimize the number of participants in sessions with additional transport costs and staff time. Several studies carried out in non-COVID-19 pandemic years have established the cost-effectiveness of using the CDI approach to deliver essential health interventions including treatment against schistosomiasis and STH [12, 13, 15–19]. As the health care system in Malawi was overwhelmed with the emergent pandemic situation, we postulate that this was a missed opportunity where the CDI approach would have been used to deliver some of the essential health services which are usually carried out by health workers. The considerable high direct costs of implementing the CDI approach, exacerbated by the unanticipated occurrence of the COVID-19 may be considered a disincentive in the short term, however, if considered as an investment, it may become cheaper in the long term due to the cumulative effect of disability-adjusted life years gained through resultant improved health services or due to the development of community capacity to handle its own health challenges. This is truer when the intervention is juxtaposed with the standard practice where it is mostly dependent on donor support which cannot always be guaranteed. Moreover, in this study for the total catchment population of 28,764 in the intervention communities, about 140 community volunteers were involved in MDA against only 25 health workers who would have been involved thereby reaching more people while freeing the health workers’ time to do other important chores. With the study mostly registering no observable differences in scores between the intervention and control arms, it would be reasonable to postulate that the CDI approach is equally capable of producing the same results as the standard approach. More than half of the total direct resources were used at the community level followed by health centre and district levels. For the cost items, most of the total resources used at all levels for both arms went to personnel related costs followed by other logistics and transportation costs. We postulate that the CDI approach is implementable at a lower cost in subsequent years if the once off cost of implementing the initial investment activities like capacity training at health centre and community levels to deal with their own health issues, would come down from 71 to 9% obtained in use of the standard approach, with an assumption that there will be no staff turnover within the health centres and communities resulting in erosion against the build up of knowledge.

The costs of indirect leveraged mostly in-kind contributions related to administrative, logistical, personnel, supplies, drugs, infrastructure, and technical expertise made by the participating institutions towards implementation of the study have not been included in this analysis. Indirect contributions are estimated to have covered 50% of the overall costs of the study. If these indirect contributions were to be included in the determination of the overall costs, then it would not be cost effective to use the CDI approach to deliver MDA campaigns against schistosomiasis and STH.

The use of the CDI approach to deliver MDA against schistosomiasis and STH raises questions about its sustainability. To ensure successful implementation and sustainability, the critical factors reported in here can be categorized into three interrelated elements as: (1) Social – related to community willingness, participation, empowerment, ownership, and commitment to CDI implementation process. (2) Policy environment—related to stakeholders’ engagement, a conducive and an enabling environment. (3) Economic considerations—related to cost effectiveness and use of incentives or rewards during implementation of CDI.

The main challenge related to implementation of the study were disruptive delays due to the COVID-19 pandemic, which challenged the original intended timing of the study as well as the budget. This included the experienced need for appropriate timing and communication with district health authorities, who had to focus more on urgent COVID-19 matters than on schistosomiasis and STH control. There was also a delay experienced by the National Schistosomiasis and STH Control Programme in delivery of drugs for the 2020 MDA campaign in all the districts due to global supply chain issues due to travel restrictions and expiry dates of allocated drugs from Merck/WHO. It is encouraging though that in this study both the health workers as providers at district and facility levels and community members as beneficiaries perceived the use of the CDI approach to deliver MDA as a welcomed and a good initiative. These sentiments and perceptions are like those also expressed in other studies within the sub-Saharan Africa region [11, 12, 14–19].

The WHO defines universal health coverage (UHC) as ‘that all people and communities have access to quality the health services where and when they need them, without financial hardship [39]. By making these essential drugs for control of schistosomiasis and STH readily available and accessible to people who need them, especially those in remote and hard-to-reach areas, MDA campaigns are a step towards attainment of the UHC goal set by the WHO [40]. Implementation of the CDI approach to deliver MDA campaigns for prevention and control of schistosomiasis and STH can also help in addressing a service delivery gap thereby making the interventions more accessible for those residing in remote and hard-to-reach areas where it is logistically challenging to distribute drugs and reach all those in need. This can be achieved by development of efficient transportation and distribution systems by the health services.

As a recommendation for future related research in Malawi, there is a need to explore more involvement and empowerment of community members in implementation of integrated NTDs control interventions such as health information, education, and communication, as well as snail control and water, sanitation, and hygiene. To enhance the coverage and sustainability of the MDA campaigns, a scaling-up of use of the CDI approach in MDA delivery may benefit from experiences and use of the ExpandNet/WHO resources through “deliberate efforts to increase the impact of successfully tested health innovations so as to benefit more people and to foster policy and programme development on a lasting basis” [4, 41].

## Conclusions

The study has demonstrated the feasibility of using the CDI approach for delivery of MDA for prevention and control of schistosomiasis and STH in Malawi. This study has also shown that there were no observed major differences in MDA coverage rates between intervention and control arms despite some challenges encountered during implementation. The findings have revealed existence of gaps in health education messages regarding control of schistosomiasis and STH. Although it was more costly to implement the interventions in the short term, the long-term benefits of using the CDI approach in delivery of MDA outweigh the investments, and both the health providers and beneficiaries perceived the interventions as good and welcome. This, therefore, could be considered as a possible way forward addressing the sustainability concern for schistosomiasis and STH control when donor support towards MDA delivery wanes.

### Supplementary Information

**Additional file 1.** A STROBE Statement—Checklist of items that should be included in reports of case-control studies.

**Additional file 2.** List of the involved health centres and villages according to their assigned study arms.

**Additional file 3.** Comparative demographic and socio-economic characteristics of study areas and populations.

## Data Availability

The datasets generated and/or analyzed during the current study are not publicly available because participants did not grant permission for public sharing of the data in our informed consent process, approved by Malawi National Health Sciences Research Committee but available from the corresponding author on reasonable request.

## Abbreviations

CDD: Community-directed distributor
CDI: Community-directed interventions
COVID-19: Coronavirus disease 2019
FGD: Focus group discussion
KAP: Knowledge attitudes and practices
KII: Key informant interview
MDA: Mass drug administration
NTD: Neglected tropical disease
STH: Soil-transmitted helminths
UHC: Universal health coverage
WHO: World Health Organization
