# Supplementary material for: Mass drug administration campaigns: comparing two approaches for schistosomiasis and soil-transmitted helminths prevention and control in selected Southern Malawi districts
Source: BMC Health Serv Res. 2024 Jan 3;24:11. doi: 10.1186/s12913-023-10489-5 (PMC10765822; doi:10.1186/s12913-023-10489-5)
Supplement: Supplementary file 2 — Additional file 2. List of the involved health centres and villages according to their assigned study arms. [file 12913_2023_10489_MOESM2_ESM.docx]

**Additional file 2:** List of the involved health centres and villages according to their assigned study arms

| District | Health centres | Villages | Assigned study arm |
| --- | --- | --- | --- |
| 1. Chiradzulu | 1. Namadzi | 1. Luna 2. Malukula 3. Masuku 4. Mitawa | 1. Intervention - CDI approach |
|  | 1. Namitambo | 1. Khumbunya 2. Likovo 3. Muhasuwa 4. Nnamala |  |
|  | 1. Maravi | 1. Kalanje 2. Likoswe 3. Mkwate 4. Ngusiche | 1. Control - Standard approach |
|  | 1. Nkalo | 1. Makalani 2. Masuso 3. Nkhupela 4. Tambala |  |
| 1. Mangochi | 1. Malombe | 1. Kadewere 2. Kamwana 3. Nalikolo 4. Ntuka | 1. Intervention - CDI approach |
|  | 1. Mtimabii | 1. Lisewa 2. Maoni 3. Mwatakata 4. Mwenyemusa |  |
|  | 1. Chilonga | 1. Chilonga 2. Kela 3. Makunula 4. Maloya | 1. Control - Standard approach |
|  | 1. Makanjira | 1. Chilawe 2. Malamia 3. Mikochi 4. Mpangama |  |
| 1. Zomba | 1. Chingale | 1. Mdalakamuyanja 2. Mlusu 3. Namwiyo 4. Ntiku | 1. Intervention - CDI approach |
|  | 1. Matiya | 1. Chaima 2. Chinkhwangwa 3. Mwaliwa 4. Naphome |  |
|  | 1. Mayaka | 1. Amiteche 2. Harry 3. Mukhwayi 4. Tambala | 1. Control - Standard approach |
|  | 1. Namadidi | 1. Issa 2. Maselema 3. Mbulukuta 4. Napwanga |  |
