# Supplementary material for: Mass drug administration campaigns: comparing two approaches for schistosomiasis and soil-transmitted helminths prevention and control in selected Southern Malawi districts
Source: BMC Health Serv Res. 2024 Jan 3;24:11. doi: 10.1186/s12913-023-10489-5 (PMC10765822; doi:10.1186/s12913-023-10489-5)
Supplement: Supplementary file 3 — Additional file 3. Comparative demographic and socio-economic characteristics of study areas and populations. [file 12913_2023_10489_MOESM3_ESM.docx]

**Additional file 3:** Comparative demographic and socio-economic characteristics of study areas and populations

| **Characteristics** | **Number (%)** | | | | | | | | | | | | | | | |
| --- | --- | --- | --- | --- | --- | --- | --- | --- | --- | --- | --- | --- | --- | --- | --- | --- |
| **District name** | **Chiradzulu** | | | | **Mangochi** | | | | **Zomba** | | | | **All Districts** | | | |
| ***Study arms*** | Intervention | | Control | | Intervention | | Control | | Intervention | | Control | | Intervention | | Control | |
| ***Health centres population*** | 65,895 (32.5) | | 60,186 (27.5) | | 67,316 (33.2) | | 69,994 (32) | | 69,332 (34.2) | | 88,661 (40.5) | | 202,543 (48.1) | | 218,841 (51.9) | |
| ***Villages population*** | 7,310 (25.4) | | 6,753 (32.9) | | 14,709 (51.1) | | 10,041 (49.0) | | 6,745 (23.4) | | 3,705 (18.1) | | 28,764 (58.4) | | 20,499 (41.6) | |
| ***Survey*** | Baseline | Follow-up | Baseline | Follow-up | Baseline | Follow-up | Baseline | Follow-up | Baseline | Follow-up | Baseline | Follow-up | Baseline | Follow-up | Baseline | Follow-up |
| ***Respondents*** | 59 (28.5) | 40 (18.0) | 67 (38.9) | 46 (28.7) | 66 (31.9) | 122 (54.9) | 63 (36.6) | 84 (52.5) | 82 (39.6) | 60 (27.0) | 42 (24.4) | 30 (18.7) | 207 (54.6) | 222 (58.1) | 172 (45.4) | 160 (41.9) |
| ***Sex***  Male  Female | 40 (28.0)  19 (29.7) | 14 (19.2)  26 (17.4) | 51 (40.8)  16 (34.0) | 11 (21.6)  35 (32.1) | 48 (33.6)  18 (28.1) | 43 (61.6)  79 (53.0) | 44 (35.2)  19 (40.4) | 29 (56.9)  55 (50.5) | 55 (38.5)  27 (42.2) | 16 (21.9)  44 (29.5) | 30 (24.0)  12 (25.5) | 11 (21.6)  19 (17.4) | 143 (69.1)  64 (30.9) | 73 (32.9)  149 (67.1) | 125 (72.7)  47 (27.3) | 51 (31.9)  109 (68.1) |
| ***Age (years)***  Mean  Range | 43.4  20-76 | 38.6  19-71 | 41.1  18-86 | 34.4  18-62 | 37.1  20-87 | 31.8  17-69 | 31.5  18-80 | 37.5  15-83 | 40.6  16-81 | 39.9  18-73 | 42.8  17-73 | 42.9  18-71 | 40.4  16-87 | 36.8  17-73 | 28.0  17-86 | 38.3  15-83 |
| ***Marital status***  Single  Married  Divorced  Widowed  Separated | 1 (1.7)  41 (69.5)  9 (15.2)  6 (10.2)  2 (3.4) | 2 (5.0)  29 (72.5)  4 (10.0)  4 (10.0)  1 (2.5) | 4 (6.0)  45 (67.2)  9 (13.4)  7 (10.4)  2 (3.0) | 2 (4.3)  34 (73.9)  5 (10.9)  2 (4.3)  3 (6.5) | 5 (7.6)  53 (80.3)  4 (6.1)  2 (3.0)  2 (3.0) | 10 (8.2)  96 (78.7)  8 (6.6)  7 (5.7)  1 (0.8) | 6 (9.5)  50 (79.4)  4 (6.3)  2 (3.2)  1 (1.6) | 4 (4.8)  62 (73.8)  11 (13.1)  5 (5.9)  2 (2.4) | 1 (1.2)  67 (81.7)  3 (3.7)  10 (12.2)  1 (1.2) | 3 (5.0)  41 (68.3)  8 (13.3)  7 (11.7)  1 (1.7) | 3 (7.1)  34 (80.9)  2 (4.8)  3 (7.1)  0 (0.0) | 0 (0.0)  24 (80.0)  1 (3.3)  3 (10.0)  2 (6.7) | 7 (3.4)  161 (77.8)  16 (7.7)  18 (8.7)  5 (2.4) | 15 (6.7)  166 (74.8)  20 (9.0)  18 (8.1)  3 (1.3) | 13 (7.5)  129 (75)  15 (8.7)  12 (7.0)  3 (1.7) | 6 (3.7)  120 (75.0)  17 (10.6)  10 (6.2)  7 (4.4) |
| ***Education***  None  Primary  Secondary  Tertiary | 5 (8.5)  34 (57.6)  20 (33.9)  0 (0.0) | 5 (12.5)  24 (60.0)  9 (22.5)  2 (5.0) | 8 (11.9)  48 (71.6)  10 (14.9)  1 (1.5) | 2 (4.3)  29 (63.0)  14 (30.4)  1 (2.2) | 23 (34.8)  33 (50.0)  9 (13.6)  1 (1.5) | 22 (18.0)  68 (55.7)  31 (25.4)  1 (0.8) | 18 (28.6)  33 (52.4)  11 (17.5)  1 (1.6) | 29 (34.5)  45 (53.6)  7 (8.3)  3 (3.6) | 17 (20.7)  51 (62.2)  13 (15.8)  1 (1.2) | 7 (11.7)  46 (76.6)  7 (11.7)  0 (0.0) | 3 (7.1)  29 (69.0)  9 (21.4)  1 (2.4) | 3 (10.0)  18 (60.0)  9 (30.0)  0 (0.0) | 35 (16.9)  118 (57.0)  42 (20.3)  2 (1.0) | 32 (14.4)  138 (62.2)  47 (21.2)  3 (1.3) | 29 (16.9)  110 (63.9)  30 (17.0)  3 (1.7) | 34 (21.2)  92 (57.5)  30 (18.7)  4 (2.5) |
| ***Occupation***  Business  Farmer  Fisher  Employed  Schooling  Unemployed  Other | 16 (27.1)  36 (61.0)  0 (0.0)  4 (6.8)  0 (0.0)  3 (5.1)  0 (0.0) | 4 (10.0)  27 (67.5)  0 (0.0)  1 (2.5)  1 (2.5)  2 (5.0)  5 (12.5) | 18 (26.9)  36 (53.7)  0 (0.0)  2 (3.0))  0 (0.0)  11 (16.4)  0 (0.0) | 9 (19.6)  26 (56.5)  0 (0.0)  2 (4.3)  0 (0.0)  2 (4.3)  7 (15.2) | 10 (15.1)  47 (71.2)  2 (3.0)  0 (0.0)  2 (3.0)  4 (6.1)  1 (1.5) | 17 (13.9)  80 (65.6)  3 (2.5)  2 (1.6)  3 (2.5)  5 (4.1)  12 (9.8) | 8 (12.7)  45 (71.4)  0 (0.0)  0 (0.0)  2 (3.2)  8 (12.7)  0 (0.0) | 9 (10.7)  56 (66.7)  1 (1.2)  5 (5.9)  2 (2.4)  5 (5.9)  6 (7.1) | 20 (24.4)  47 (57.3)  6 (7.3)  3 (3.7)  0 (0.0)  4 (4.9)  2 (2.4) | 12 (20.0)  44 (73.3)  0 (0.0)  0 (0.0)  0 (0.0)  3 (5.0)  1 (1.7) | 9 (21.4)  25 (59.5)  0 (0.0)  2 (4.8)  0 (0.0)  6 (14.3)  0 (9.0) | 5 (16.7)  23 (76.7)  0 (0.0)  0 (0.0)  0 (0.0)  1 (3.3)  1 (3.3) | 46 (22.2)  130 (62.8)  8 (3.9)  7 (3.4)  2 (1.0)  11 (5.3)  3 (1.4) | 33 (14.9)  151 (67.6)  3 (1.3)  3 (1.3)  4 (1.8)  10 (4.5)  18 (8.1) | 35 (20.3)  106 (61.6)  0 (0.0)  4 (2.3)  2 (1.2)  25 (14.5)  0 (0.0) | 23 (14.4)  105 (65.6)  1 (0.6)  7 (4.4)  2 (1.2)  8 (5.0)  14 (8.7) |
